# KRAS and TP53 mutations in inflammatory bowel disease-associated colorectal cancer: a meta-analysis

**DOI:** 10.18632/oncotarget.14549

**Published:** 2017-01-07

**Authors:** Lijun Du, John J. Kim, Jinhua Shen, Binrui Chen, Ning Dai

**Affiliations:** ^1^ Department of Gastroenterology, Sir Run Run Shaw Hospital, School of Medicine, Zhejiang University, Hangzhou, China; ^2^ Division of Gastroenterology, Loma Linda University Medical Center, Loma Linda, USA; ^3^ Department of Gastroenterology, Affiliated Hospital of Shaoxing University, Shaoxing, China

**Keywords:** KRAS, TP53, mutation, inflammatory bowel disease, colorectal cancer

## Abstract

Although KRAS and TP53 mutations are common in both inflammatory bowel disease-associated colorectal cancer (IBD-CRC) and sporadic colorectal cancer (S-CRC), molecular events leading to carcinogenesis may be different. Previous studies comparing the frequency of KRAS and TP53 mutations in IBD-CRC and S-CRC were inconsistent. We performed a meta-analysis to compare the presence of KRAS and TP53 mutations among patients with IBD-CRC, S-CRC, and IBD without dysplasia. A total of 19 publications (482 patients with IBD-CRC, 4,222 with S-CRC, 281 with IBD without dysplasia) met the study inclusion criteria. KRAS mutation was less frequent (RR=0.71, 95%CI 0.56-0.90; *P*=0.004) while TP53 mutation was more common (RR=1.24, 95%CI 1.10-1.39; *P*<0.001) in patients with IBD-CRC compared to S-CRC. Both KRAS (RR=3.09, 95%CI 1.47-6.51; *P*=0.003) and TP53 (RR=2.15, 95%CI 1.07-4.31 *P*=0.03) mutations were more prevalent in patients with IBD-CRC compared to IBD without dysplasia. In conclusion, IBD-CRC and S-CRC appear to have biologically different molecular pathways. TP53 appears to be more important than KRAS in IBD-CRC compared to S-CRC. Our findings suggest possible roles of TP53 and KRAS as biomarkers for cancer and dysplasia screening among patients with IBD and may also provide targeted therapy in patients with IBD-CRC.

## INTRODUCTION

Inflammatory bowel disease (IBD) is an idiopathic, chronic relapsing inflammatory disorder of the gastrointestinal tract, comprising of ulcerative colitis (UC) and Crohn's disease (CD). Patients with IBD have an increased risk of developing colorectal cancer (CRC) as early as 8-10 years after the diagnosis [1, 2]. A meta-analysis demonstrates a 4.5-fold increase in risk of CRC in patients with IBD compared to the general population, and CRC accounts for approximately 15% of all deaths in patients with IBD [3–5].

Inflammation and genetic instability contribute to the development of IBD-associated CRC (IBD-CRC) [6–8]. Series of alterations in oncogenes and tumor suppressor genes typically observed in sporadic CRC (S-CRC) are also important in the carcinogenesis of IBD-CRC. KRAS, a proto-oncogene, and TP53, a tumor suppressor gene, are strongly implicated in S-CRC. Mutations in KRAS and TP53 have demonstrated strong association with tumor progression in S-CRC [9–11]. Notably, KRAS mutational status determines the efficacy of epidermal growth factor receptor (EGFR) inhibitor, a potent therapy for patients with CRC in clinical practice [12, 13]. However, involved gene sequences and mutation frequencies of KRAS and TP53 may differ between S-CRC and IBD-CRC [14]. For example, TP53 mutations occur in the early stages of oncogenesis in IBD-CRC compared to late stages in S-CRC [15–17]. Furthermore, the loss of heterozygosity for TP53 is associated with progression of dysplasia, and TP53 mutation can also occur before the loss of heterozygosity in patients with UC. In contrast, KRAS mutation occurs in the later stages and less frequently in the oncogenesis of IBD-CRC compared to S-CRC [16, 18, 19].

Understanding differences in tumorigenesis between IBD-CRC and S-CRC may provide opportunity for targeted therapy for patients with IBD-CRC. In addition, evaluation of KRAS and TP53 mutation status can be used as potential biomarkers for dysplasia and cancer screening in patients with IBD. The aim of the meta-analysis was to compare the frequency of KRAS and TP53 mutation among patients with IBD-CRC, S-CRC, and IBD without dysplasia.

## RESULTS

### Literature search and description of included studies

**Figure 1:**
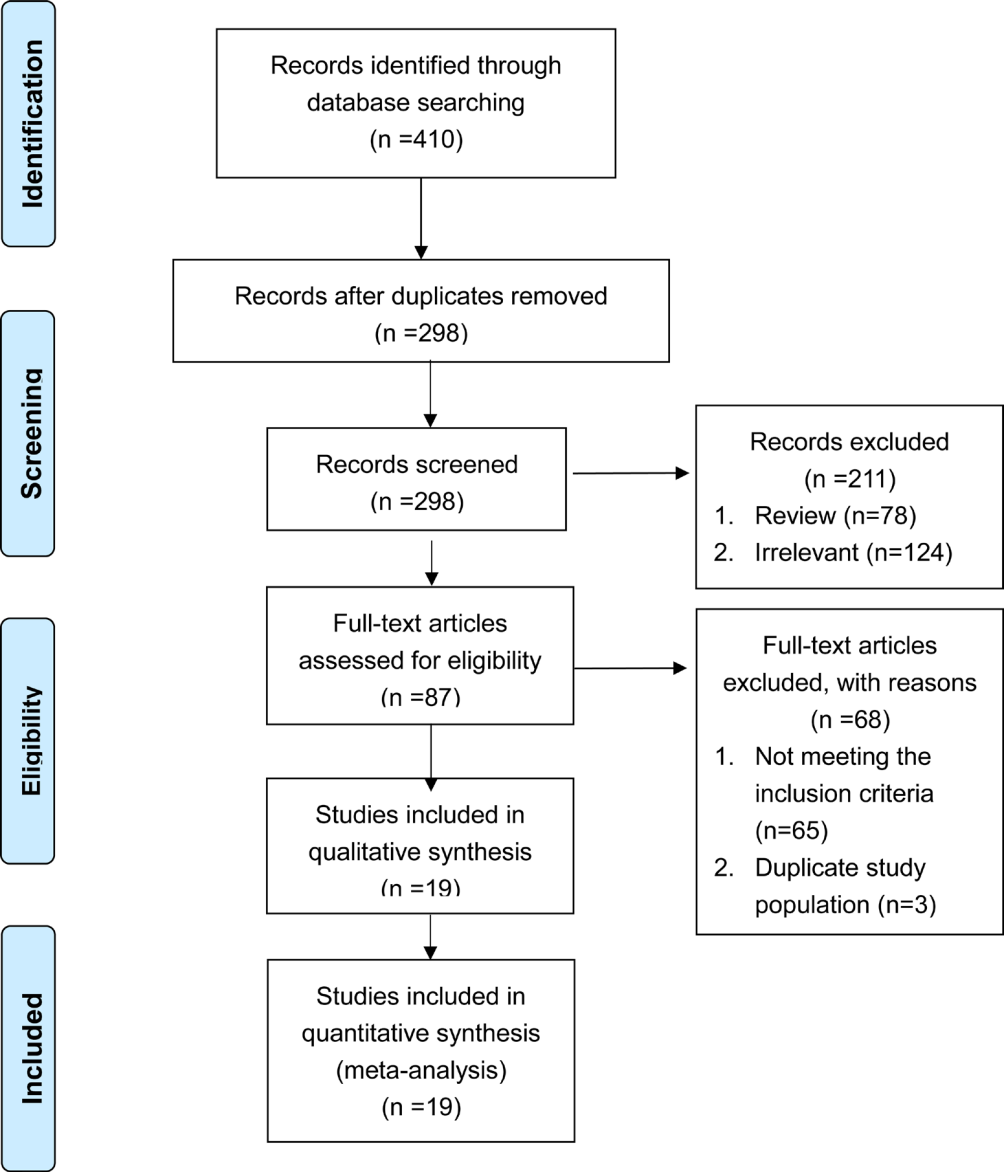
Flow chart of the selection process for the included studies

An initial search retrieved 410 published studies. After a careful selection process, 17 case-control studies and two cohort studies were included in this meta-analysis (Figure 1) [20–38]. Quality scores of the 19 selected studies ranged from seven to nine indicating moderate to high quality. All studies were considered acceptable for inclusion in the meta-analysis. Quality scores of the included studies were summarized in Table 1.

**Table 1:** Demographic and clinical characteristics of included studies

Study	Country	Cases	Controls	Analytical Method	Mutation Gene	Mutation Site	NOS
Burmer 1991	UK	UC-CRC	S-CRC	PCR	KRAS	Codon 12	7
Bell 1991	USA	UC-CRC or dysplasia	S-CRC	PCR	KRAS	Codon 12	9
Taylor 1993	UK	IBD-CRC	S-CRC	IHC positive	TP53	NA	9
Rashid 1997	USA	CD associated adenocarcinomas	S-CRC	≥50% IHC positive/PCR	TP53/KRAS	NA/codon 12, 13	7
Lashner 1999	USA	UC-CRC	UC	≥5% IHC positive	TP53	NA	7
Walsh 1999	USA	UC-CRC or dysplasia	S-CRC	IHC positive	TP53	NA	9
Reeves 2004	UK	IBD-CRC	S-CRC	PCR	TP53/KRAS	NA/codon 12	7
Maia 2005	Portugal	IBD-CRC or dysplasia	IBD	PCR-SSCP	TP53	Exon 6, 7	7
Bossard 2007	France	IBD-CRC	IBD	PCR	KRAS	Codon 12, 13	7
Nathanson 2008	USA	CD dysplasia	CD	Dark-brown IHC stain	TP53	NA	7
Laurent 2011	France	IBD-CRC	S-CRC, IBD	≥10% IHC positive	TP53	NA	7
Sanchez 2011	USA	UC-CRC	S-CRC	PCR	TP53/KRAS	Exon 4, 8/ codon 12, 13	9
Olaru 2012	USA	IBD-CRC	S-CRC	PCR	KRAS	NA	7
Shivakumar 2012	India	IBD-CRC	S-CRC, UC	PCR	TP53/KRAS	Exon 4-8/ codon 12, 13	7
Kisiel 2013	USA	IBD-CRC	IBD	PCR	TP53/KRAS	NA	9
Ottessen 2015	Norway	UC-CRC	UC	≥5% IHC positive	TP53	NA	7
Johnson 2016*	USA	IBD-CRC	IBD	PCR	KRAS	NA	9
Lennerz 2016	USA	CD-CRC	S-CRC	PCR	KRAS	NA	7
Yaeger 2016	USA	IBD-CRC	S-CRC	PCR	TP53/KRAS	NA	7

IBD: inflammatory bowel disease; UC: ulcerative colitis; CD: Crohn’s disease; CRC: colorectal cancer; PCR: polymerase chain reaction; SSCP: single-strand conformation polymorphism; S-CRC: sporadic colorectal cancer; NOS: Newcastle-Ottawa Scale.

*The study by Johnson et al 2016 contained two independent phases, and the data of one cohort phase was first presented in 2014

A total of 482 patients with IBD-CRC, 4,222 with S-CRC, and 281 with IBD without dysplasia were included in our analysis. Twelve studies were conducted in the U.S., which is a high-prevalence area for IBD. Six studies were conducted in Europe, and one study was conducted in India [39–41]. The mean sample size of patients with IBD-CRC in all the studies was 24 (range 6 to 47). Thirteen studies described TP53 mutation, and 12 studies reported KRAS mutation, respectively. The most frequently reported mutational sites of TP53 and KRAS were exon 4-8 and exon 2, respectively. Codon 12 located in exon 2 had the highest mutation rate of KRAS. The demographic and clinical characteristics of included studies were shown in Table 1.

### KRAS mutation in IBD-CRC

**Figure 2:**
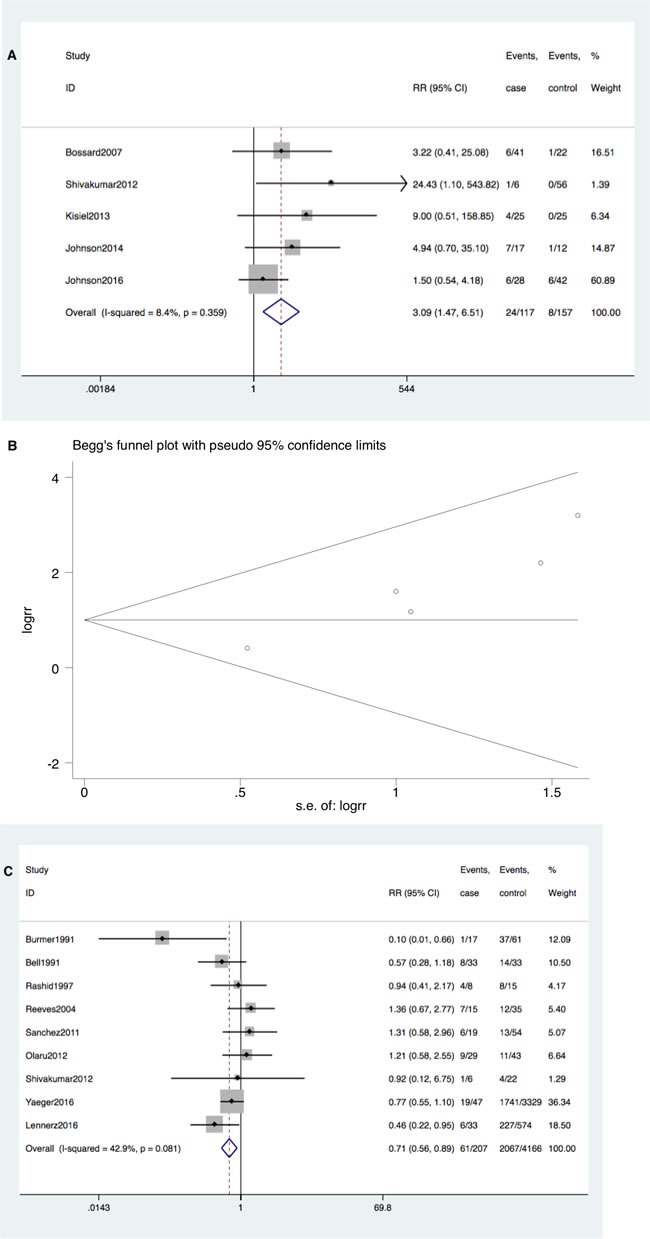
**A. Forrest plot of risk ratio (RR) for KRAS mutation comparing IBD-CRC and IBD without dysplasia**. **B**. Begg's funnel plot of enrolled studies. **C**. Forrest plot of RR for KRAS mutation comparing IBD-CRC and S-CRC. **D**. Begg's funnel plot of enrolled studies.

KRAS mutation occurred more frequently in patients with IBD-CRC compared to IBD patients without dysplasia (RR = 3.09; 95%CI 1.47-6.51, *P* = 0.003). Neither significant heterogeneity (I^2^ = 8.4%, *P* = 0.36) nor publication bias (Begg's test *P* = 0.09, Egger's test *P* = 0.08) was detected (Figure 2A and 2B).

However, KRAS mutation occurred less commonly in IBD-CRC compared to S-CRC (RR = 0.71; 95% CI: 0.56-0.90, *P* = 0.004). Neither significant heterogeneity (I^2^ = 42.9%, *P* = 0.08) nor publication bias (Begg's test *P* = 0.60, Egger's test *P* = 0.65) was detected (Figure 2C and 2D).

### TP53 mutation in IBD-CRC

**Figure 3:**
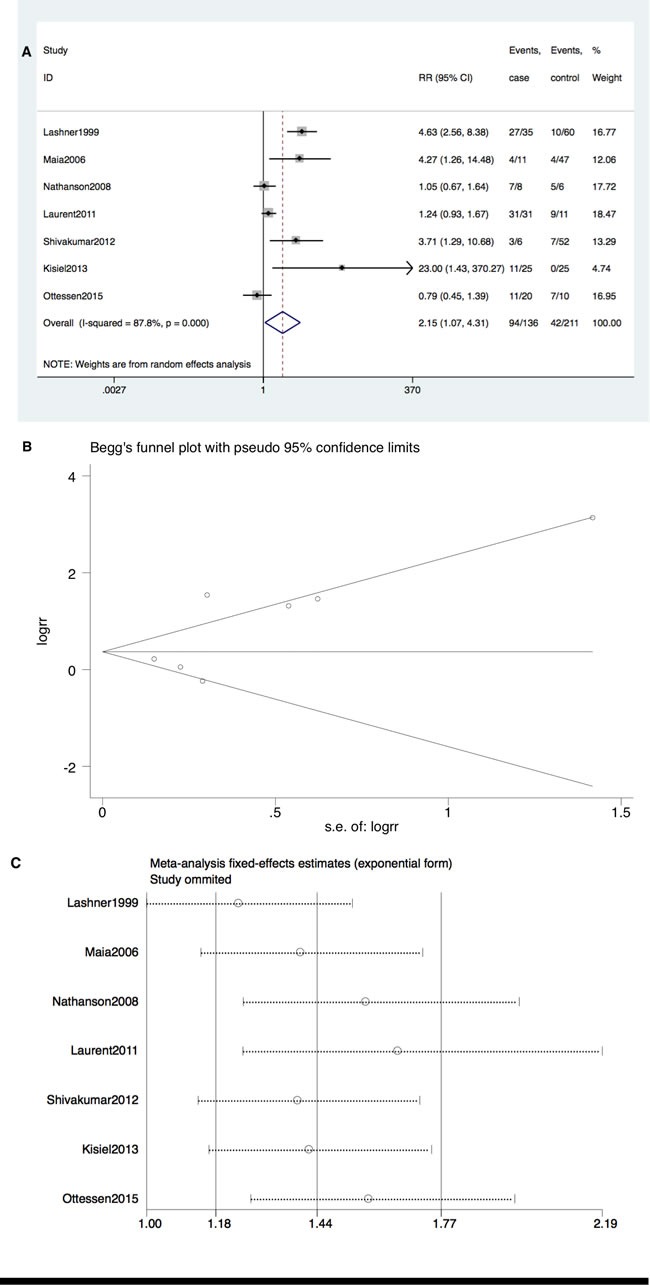
**A. Forrest plot of RR for TP53 mutation comparing IBD-CRC and IBD without dysplasia**. **B**. Begg's funnel plot of enrolled studies. **C**. sensitivity analysis.

TP53 mutation also occurred more frequently in patients to IBD-CRC compared with IBD patients without dysplasia (RR = 2.15; 95% CI: 1.07-4.31, *P* = 0.03). Significant heterogeneity among studies (I^2^ = 87.8%, *P* < 0.001) but no publication bias (Begg's test P = 0.368, Egger's test *P* = 0.131) was detected (Figure 3A and 3B). However, no single publication was found to be significantly biasing the results using sensitivity analysis (Figure 3C).

**Figure 4:**
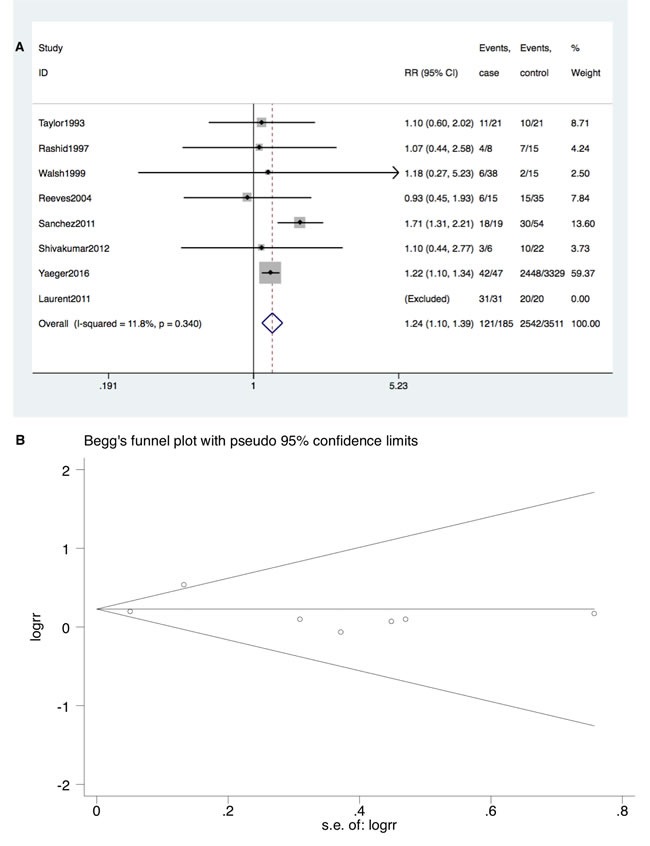
**A. Forrest plot of RR for TP53 mutation comparing IBD-CRC and S-CRC. B**. Begg's funnel plot of enrolled studies.

Furthermore, TP53 mutation occurred at a higher rate in IBD-CRC compared to S-CRC (RR = 1.24; 95% CI: 1.10-1.39, *P* < 0.001). Neither significant heterogeneity (I^2^ = 11.8%, *P* = 0.34) nor publication bias (Begg's test *P* = 0.23, Egger's test *P* = 0.91) was detected (Figure 4A and 4B).

### Carcinogenesis in IBD patients with TP53 mutation

**Figure 5:**
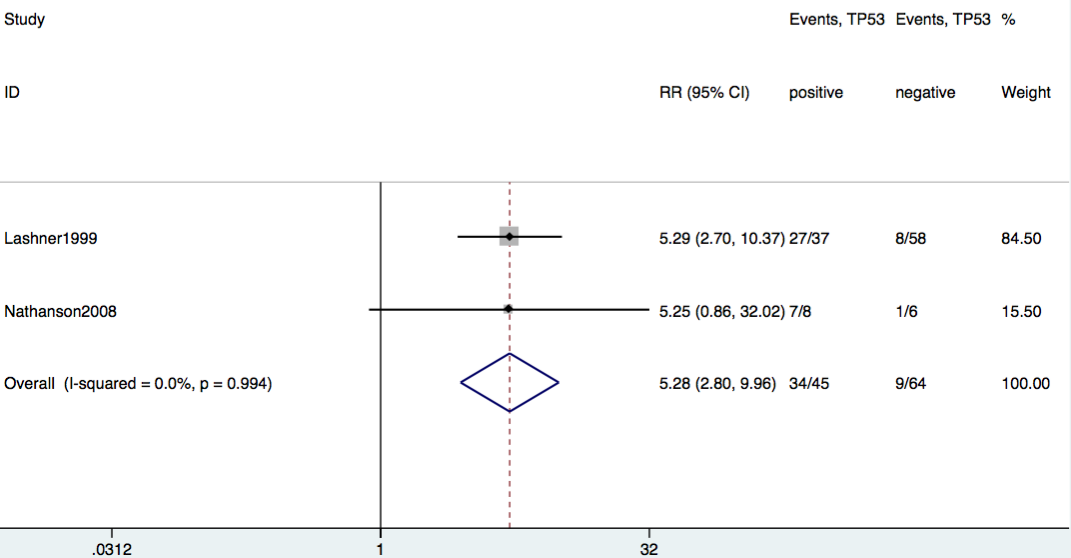
Forrest plot of RR for developing CRC in patients with IBD with or without TP53 mutation

Of the 19 studies, only two studies reported the incidence of IBD-CRC as an outcome among IBD patients with or without TP53 mutation. As shown in Figure 5, there was a significant association between incidence of IBD-CRC and TP53 mutation among patients with IBD without dysplasia (RR = 5.28; 95% CI: 2.80-10.0, *P* < 0.001).

## DISCUSSION

To our knowledge, this is the first meta-analysis comparing the frequency of KRAS and TP53 gene mutations among patients with IBD-CRC, S-CRC, and IBD without dysplasia. The meta-analysis demonstrated that TP53 mutation is more frequent while KRAS mutation is less prevalent in patients with IBD-CRC compared to S-CRC. Furthermore, both TP53 and KRAS mutations are more common in patients with IBD-CRC compared to IBD without dysplasia. Finally, IBD patients with TP53 mutation are more likely to develop IBD-CRC.

Observational studies have demonstrated no appreciable difference in clinical outcome including tumor recurrence, disease-free survival, and overall survival among patients with IBD-CRC compared to S-CRC patients [42, 43]. Possible reasons for the lack of difference in outcome despite distinct demographic and clinical patient characteristics may be related to utilizing conventional CRC therapy for patients with IBD-CRC, without accounting for the difference in molecular pathway. However, there are a number of important differences in carcinogenesis and outcome among patients with IBD-CRC and S-CRC, which may provide opportunity for targeted therapy and surveillance. Specifically, KRAS and TP53 status have been shown to be associated with progression-free survival and overall survival in patients with CRC [44, 45]. Previous molecular studies have highlighted the differences between KRAS and TP53 in the prevalence and onset of molecular events in between IBD-CRC and S-CRC [46]. Although KRAS mutations commonly have been reported among patients with S-CRC (18%-61%) and IBD-CRC/dysplasia (6%-50%) [21, 23, 33], KRAS mutation may occur later in IBD-CRC/dysplasia compared to S-CRC. Furthermore although TP53 mutations also are common in S-CRC (13%-100%) and IBD-CRC/dysplasia (16%-100%), TP53 mutation may occur early in IBD-CRC/dysplasia compared to S-CRC [25, 30]. However, a wide variation in mutational rate have been reported between different studies likely related to the differences in the study population.

The results of this meta-analysis provides support for possible genomic differences between IBD-CRC and S-CRC. Furthermore, the anatomic location of the tumors as well as the frequency and sequence of the molecular events differ between IBD-CRC and S-CRC according to previous evidence. IBD-CRC develops in chronically inflamed mucosa typically from absence of dysplasia, to indefinite dysplasia, to low-grade dysplasia, to high-grade dysplasia, and to carcinoma. This sequence is different from S-CRC that generally develops from a polypoid adenoma [15, 47]. The differences in expression of mutational KRAS and TP53 genes in IBD-CRC may account for the phenotypical differences of the two groups of CRC.

Some studies demonstrated that KRAS gene mutation is less frequent in IBD-CRC compared to S-CRC, consistent with the result of the meta-analysis [48, 49]. KRAS is a membrane bound proto-oncogene and functions as GDP-GTP-regulated binary on-off switch that regulates cytoplasmic signaling pathways and controls wide range of physiologic cellular processes [50]. KRAS is a key downstream effector of EGFR, and permanent activation of KRAS as a result of mutation causes cells to grow without exogenous stimulation and drives tumor initiation [51, 52]. Thus, tumors with mutant KRAS tend to be unresponsive to anti-EGFR therapy with increased risk of relapse and death compared with patients who have tumors characterized by a wide-type KRAS status [53]. A meta-analysis that includes patients with metastatic CRC demonstrated that KRAS mutations are highly specific, negative predictors of anti-EGFR therapy [54]. Given that KRAS status is a robust predictor of clinical response in multiple clinical trials, National Comprehensive Cancer Network (NCCN) Clinical Practice Guidelines have recommended genotyping KRAS status upon diagnosis of stage IV disease and also prior to administering EGFR-targeted treatment [55, 56]. Furthermore, presence of codon 12 mutations, the most common KRAS mutation, is associated with worsening relapse-free survival across all stages of CRC [57]. Therefore, KRAS mutation status is an important predictor of therapeutic efficacy in CRC.

Tumor suppressor genes are important guardians preventing expansion of mutant cells. Thus, the genetic abnormalities are associated with poor prognosis in patients with S-CRC. The important role of wide-type TP53, a tumor suppressor gene, is to halt the progression of cell cycle if DNA damage has occurred. Thus, a mutant p53 protein shows loss of growth-inhibitory function [58]. Furthermore, the wild-type p53 proteins have an extremely short half-life, whereas mutated p53 protein products are relatively stable and can be used as markers of mutated TP53 [59, 60]. Therefore, immunohistochemistry (IHC) can be used to determine the expression and location of p53 proteins that have accumulated in the cell nuclei of cancer tissues. Previous studies have shown that IHC and polymerase chain reaction (PCR) have a 95% concordance of measuring genetic mutations or protein accumulation in the nucleus [61]. A possible reason for the heterogeneity among studies comparing TP53 between IBD-CRC and IBD without dysplasia may be related with differing methodologies for evaluating TP53. Although determining TP53 status, unlike KRAS status, was not recommended by the NCCN as a prognostic predictor, the high frequency of TP53 mutations observed in many sequenced cancers highlight the important role of TP53 in the inhibition of cancer development [62, 63]. For example, TP53 mutation or loss of heterozygosity is associated with a higher cancer stage at presentation, a higher rate of recurrence, and a higher mortality [64–66]. Burmer and colleagues [67] found that TP53 loss of heterozygosity was detected in 6% of the biopsy specimens of UC patients without dysplasia, 9% with indefinite dysplasia, 33% with low grade dysplasia, 63% with high grade dysplasia and 85% with cancer. The results of our meta-analysis indicate that both KRAS and TP53 mutations occur more frequently in patients with IBD-CRC compared to IBD without dysplasia, consistent with previous studies. Furthermore, we found that TP53 mutation is associated with more advanced stage (Duke's Class A, B, C) in patients with IBD-CRC (data not shown). Given our findings, TP53 status may potentially be used as a biomarker to improve cancer and dysplasia screening among patients with IBD.

There are limitations to our meta-analysis mainly related to the small number of published studies in full-text and English including some studies with a small sample size leading to the possibility of bias. For example, our findings demonstrating increased risk of CRC among IBD patients with TP53 mutation compared to those without TP53 mutation will require validation, given the inclusion of only two studies for the analysis. In addition, heterogeneity among the studies including study design and methods assessing KRAS and TP53 mutation may potentially affect the results of the meta-analysis.

In conclusion, our meta-analysis suggests that IBD-CRC and S-CRC may have different molecular pathways given the higher prevalence of TP53 but lower prevalence of KRAS mutation in the patients with IBD-CRC compared to S-CRC. Our findings suggest possible roles of TP53 and KRAS as candidate biomarkers for cancer and dysplasia screening among patients with IBD. Furthermore, elucidating the molecular pathway unique to IBD-CRC may provide potential targeted therapy. Further clinical studies are needed to validate our findings.

## MATERIALS AND METHODS

### Search strategy

A comprehensive literature search was conducted using Pubmed, EMBASE, and The Cochrane Library databases with an end date of June 2016. The main search strategies were as follows: “KRAS OR TP53” AND “mutation OR mutational analysis” AND “inflammatory bowel disease OR ulcerative colitis OR Crohn's disease” AND “colorectal OR colon OR intestinal” AND “cancer OR adenocarcinoma OR carcinoma OR neoplasm”.

### Study selection

Studies were required to meet the following inclusion criteria: (1) case-control or cohort studies; (2) provided a confirmed diagnosis of IBD-CRC in humans; (3) explicitly reported the detection methods for KRAS and TP53 alterations including PCR-SSCP, DNA sequencing, or other specific approaches for identifying gene mutation; and (4) written in English. In addition, reviews, animal studies, case reports, and studies lacking relevant data were excluded. Two investigators (L.D., B.C.) independently read the titles and abstracts of candidate studies. Afterwards, the two investigators analyzed the full texts of selected studies to determine whether the studies met the inclusion criteria. For duplicate studies based on identical or overlapping patient populations, the most recent or complete study was included in this meta-analysis. Any disagreement was resolved by a third investigator (N.D.).

### Data extraction and quality evaluation

Two investigators (L.D., J.S.) independently extracted the data including the first author, publication year, geographic location, analytical method (protein/gene), cut-off values, and detected exons. The quality of the studies were assessed by using the Newcastle-Ottawa Scale (NOS) based on three perspectives: selection, comparability, and ascertainment of outcome [68]. Full score is nine stars, and studies with more stars were considered to be of higher quality.

### Study endpoints and statistical analysis

The mutational status of KRAS and TP53 in IBD-CRC compared to IBD without dysplasia and S-CRC based on data from case-control and cohort studies were assessed. The endpoints of interest were expressed by risk ratios (RRs) with 95% CI. If the study was homogeneous (I^2^ < 50%), the fixed-effects model was used; otherwise (I^2^ > 50%), the random-effects model was chosen [69]. A *P*-value < 0.05 was considered statistically significant. If the study was heterogeneous, a sensitivity analysis was performed to examine the impact on the overall results. Publication bias was assessed by Egger's and Begg's test. All data were analyzed with Stata 12.0.
